# CBCT‐based assessment of the anatomic relationship between maxillary sinus and upper teeth

**DOI:** 10.1002/cre2.451

**Published:** 2021-05-22

**Authors:** Tobias Regnstrand, Andres Torres, Eline Petitjean, Paul Lambrechts, Daniel Benchimol, Reinhilde Jacobs

**Affiliations:** ^1^ Section of Oral Diagnostics and Surgery, Division of Oral Diagnostics and Rehabilitation, Department of Dental Medicine Karolinska Institutet Huddinge Sweden; ^2^ OMFS‐IMPATH Research Group, Department of Imaging and Pathology, Faculty of Medicine Catholic University of Leuven Leuven Belgium; ^3^ Department of Oral Health Sciences, Endodontology, KU Leuven & Dentistry University Hospitals Leuven Leuven Belgium

**Keywords:** anatomy, canine, cone‐beam computed tomography, maxillary sinus, molar, premolar

## Abstract

**Objectives:**

To describe the relationship between maxillary sinus (MS) and upper teeth based on cone beam computed tomographic scans (CBCT).

**Materials and methods:**

Based on CBCT maxillary imaging data of 147 patients, distance between MS and apices of canines and posterior maxillary teeth were assessed. Distances between tooth roots and sinus were classified into three groups: distant (>2 mm), close (<2 mm) or in contact with MS. Teeth with apical lesions and uncommon root configurations were excluded.

**Results:**

In total, 1075 teeth of maxillary canines, upper premolars and upper molars were included in this study. Teeth most often in contact with MS were the second (89%) and first (81%) maxillary molar without any significant difference (*p* = 0.19). Roots most often in contact with MS were the mesiobuccal and distobuccal root of the second molar (85% and 76%; *p* = <0.01) followed by the palatal root of the first molar (73%). A fifth of the upper canines are situated less than 2 mm from MS.

**Conclusions:**

More than four out of five upper molars (first and second) are in a close relationship to the MS. Knowledge of the anatomical relationship between posterior maxillary teeth and the MS is important for diagnosis and treatment in this area.

## 1 INTRODUCTION

The oral cavity is situated in a close relationship with several other anatomic structures of importance in the head ‐ neck area. An improved understanding of the relationship between canines and posterior maxillary teeth (PMT) and maxillary sinus (MS) is of value for dentists and oral surgeons who regularly perform treatments in this area. Posterior teeth in the upper jaw may have a close proximity to the MS (Hu et al., 2019; Junqueira et al., 2020; Kilic et al., 2010; Nino‐Barrera et al., 2018; Shahbazian et al., 2014; Shokri et al., 2014; Zhang et al., 2019). Considering a close proximity between the apices of PMT and the MS, a dental infection may spread into the MS. Approximately 40% of unilateral maxillary sinusitis cases have a dental origin whereas the number is 24% for bilateral maxillary sinusitis cases (Vestin Fredriksson et al., 2017). When maxillary sinusitis has an odontogenic etiology, specific dental treatment such as endodontic treatment or extraction may be required. Some examples of dental causes are apical infections and adult periodontal breakdown (Vestin Fredriksson et al., 2017). The likelihood for a sinusitis of dental origin increases due to a closer relationship between the infected root and the MS (Mehra & Murad, 2004). Therefore, a thorough knowledge of the relationship between upper canines, posterior teeth and MS is of great value in diagnosing sinusitis with a probable dental cause. For several dental treatments such as endodontics and extractions, the relationship of PMT and MS is necessary to take into consideration. If the root apex is in close proximity to the MS, the periapical inflammation may involve the Schneiderian membrane (Nunes et al., 2016). Also, in treatment planning of apical surgery, knowledge of the relationship between apex and MS is of importance. The thin bone between the roots of the PMT and the MS may fracture during tooth extractions, potentially causing an oroantral communication. The upper first molar is the tooth most frequently involved in oroantral communications, with an occurrence of 1 in 150 extractions (Punwutikorn et al., 1994). An untreated or unhealed oroantral communication may cause chronic sinusitis. Several studies have reported on the relation between upper teeth and MS (Hu et al., 2019; Junqueira et al., 2020; Kilic et al., 2010; Nino‐Barrera et al., 2018; Shahbazian et al., 2014; Shokri et al., 2014; Zhang et al., 2019). Yet, few previous studies have fully described and fully quantified the relationship of all upper teeth with the MS. Previous studies has assessed the relationship between PMT and MS, not including the relationship between canines and MS (Estrela et al., 2016; Junqueira et al., 2020; Kilic et al., 2010; Nino‐Barrera et al., 2018; von Arx et al., 2014; Zhang et al., 2019). The aim of this study is to describe the relationship between MS and upper teeth in CBCT.

## 2 MATERIALS AND METHODS

A retrospective analysis of cone beam computed tomography (CBCT) scans was carried out. The study was approved by the clinical trial center and the ethical committee of the Catholic University of Leuven and the University Hospital of Leuven (s58691). A total of 380 CBCT scans of patients referred to the University Hospital of Leuven were assessed. The CBCT scans were taken between January 1, 2013 and July 1, 2013. Scans were included when the entire MS floor of at least one MS was visible. Only the maxillary canines, premolars and molars in the upper jaw were assessed. These teeth and their periapical region had to be completely visible in the volume to be included. Scans containing no teeth, primary teeth, permanent teeth with open apices, maxillary implants, sinus augmentations, oroantral communications and scans of low quality were excluded. Scans taken after maxillofacial injury or orthognathic surgery were excluded. In addition, impacted teeth, teeth presenting severe periodontitis (bone loss exceeding more than half of the root length), teeth with uncommon root configuration (teeth with uncommon number of roots for that specific tooth type) and teeth with periapical or periradicular lesions were excluded.

CBCT scans were taken using the 3D Accuitomo 170 device (3D Accuitomo, J. Morita, Kyoto, Japan) operating at 90 kV and 5 mA with an exposure time of 17.5 s. Field of view (FOV) were 6 x 6, 8 x 8, 10 x 10, 14 x 14, 14 x 10, 14 x 10, and 17 x 17 cm depending on the case. Voxel sizes in the CBCT exams varied between 0.125 and 0.250 mm. The scans were evaluated using the i‐Dixel 2.0 software (J. Morita USA, Inc., Irvine, CA) on a 30″ monitor with a resolution of 2560 × 1600 pixels (Dell 3008WFP, Dell, Inc., Round Rock, TX) in a dimmed room. The slice thickness varied between 0.5 and 1 mm. Measurement of the shortest distance between the radiological apex and the MS floor were performed in slices reformatted to align the apical part of the root axis vertically in the sagittal and coronal views.

**FIGURE 1:**
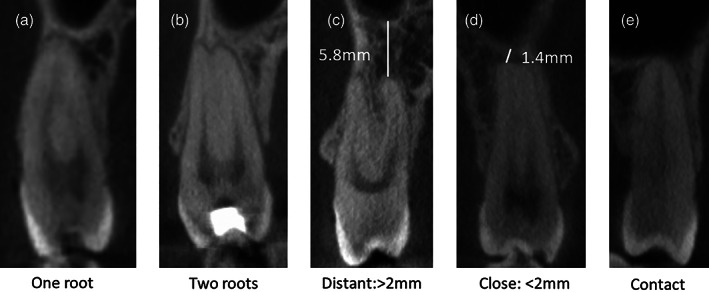
One root (a), tooth is defined as two rooted if the apices are separated (b). Measurements showing a distant (c), close (d), and contact (e) relationship between the apices and maxillary sinus

Teeth with the following root configurations were included in the study; canine: single rooted, 1st premolar: single rooted/two rooted; buccal and palatal, 2nd premolar: single rooted/two rooted; buccal and palatal, 1st molar: three rooted; mesiobuccal (MB), distobuccal (DB) and palatal (P), 2nd molar: three rooted; MB, DB and P and 3rd molars: single rooted/three rooted; MB, DB and P root. A tooth presenting separate roots was recorded if there was a division between the radiological apices (Figure 1). The relationship between the MS and the apices of the maxillary teeth were divided into three groups:

*Distant*: >2 mm between the radiological apex and the MS floor.

*Close*: <2 mm between radiological apex and the MS floor, no fusion between lamina dura of the root and the cortical border of the MS floor.

*Contact*: The lamina dura of the radiological apex is fused with the cortical border of MS floor. The apex can be at the same level or protruding into the floor of the MS. The present study groups the measures in these three groups for a more comprehensive and clinically relevant grouping. Two observers, senior postgraduate students in endodontics, screened all CBCT scans independently. When disagreement occurred regarding the distance to MS, the observers looked at the case together and reached a consensus.

### 2.1 Statistical analysis

Statistical analyses were performed with S‐plus 8.0 for Linux (Tibco software, Palo Alto, CA). The data were first analyzed descriptively for mean values and percentage distribution. To detect significant differences, *p*‐values were calculated by means of a generalized linear mixed model for binary outcomes using a logit‐link. A univariate separate model was built for the different sinus relations (distant, <2 mm, touching, protruding, and touching or protruding) separately. Explanatory variables were tooth category, root category and age category.

Comparison between the root categories were assessed for each tooth type separately. The difference between age categories were assessed once for each tooth category and once for each combination of root and tooth category.

In addition, the occurrence of roots with a close sinus relation was compared between the genders.

## 3 RESULTS

**FIGURE 2:**
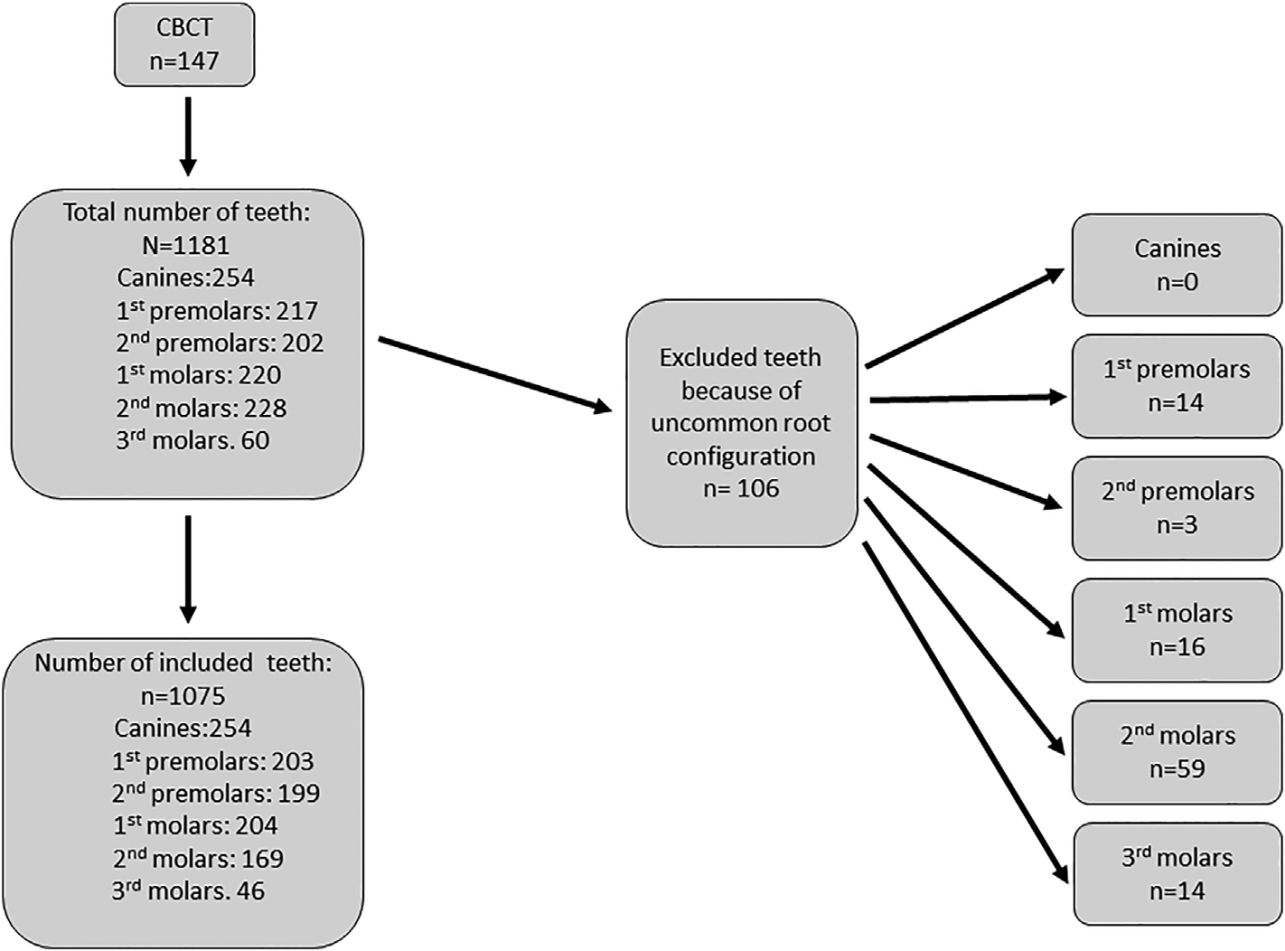
Overview of excluded teeth with uncommon root configuration divided into tooth types

After initial assessment, a total of 147 CBCT scans containing 258 sinuses and 1075 teeth met the inclusion criteria. The volumes were classified according to the FOV in small <8 x 8cm (*n* = 22), medium = 8 x 8cm (*n* = 83), and large >8 x 8cm (*n* = 42). A total of 106 teeth were excluded because of uncommon root configuration (Figure 2).

Data were collected from CBCT examinations of 147 patients; males *n* = 59 (40%), females *n* = 88 (60%) with a mean age of 46 years (range 15–84 years). Age distribution was ≤20 years *n* = 5, 21–40 years *n* = 51, 41–60 years *n* = 59, >60 years *n* = 32. A total of 1181 teeth were assessed, and after exclusion of teeth with uncommon root anatomy, 1075 teeth (canines *n* = 254, 1st premolars *n* = 203, 2nd premolars *n* = 199, 1st molars *n* = 204, 2nd molars *n* = 169, 3rd molars *n* = 46) were included in the study. In total, 1984 roots were analyzed. The number of excluded teeth because of uncommon root configuration was for canines *n* = 0, 1st premolars *n* = 14, 2nd premolars *n* = 3, 1st molars *n* = 16, 2nd molars *n* = 59 and 3rd molars *n* = 14 (Figure 2). Among the evaluated teeth, 24% were canines, 19% 1st premolars (45% single rooted and 55% two rooted), 19% 2nd premolars (92% single rooted and 8% two rooted), 19% 1st molars, 15% 2nd molars, 4% 3rd molars (61% single rooted and 39% three rooted).

### 3.1 Relationship to maxillary sinus

**FIGURE 3:**
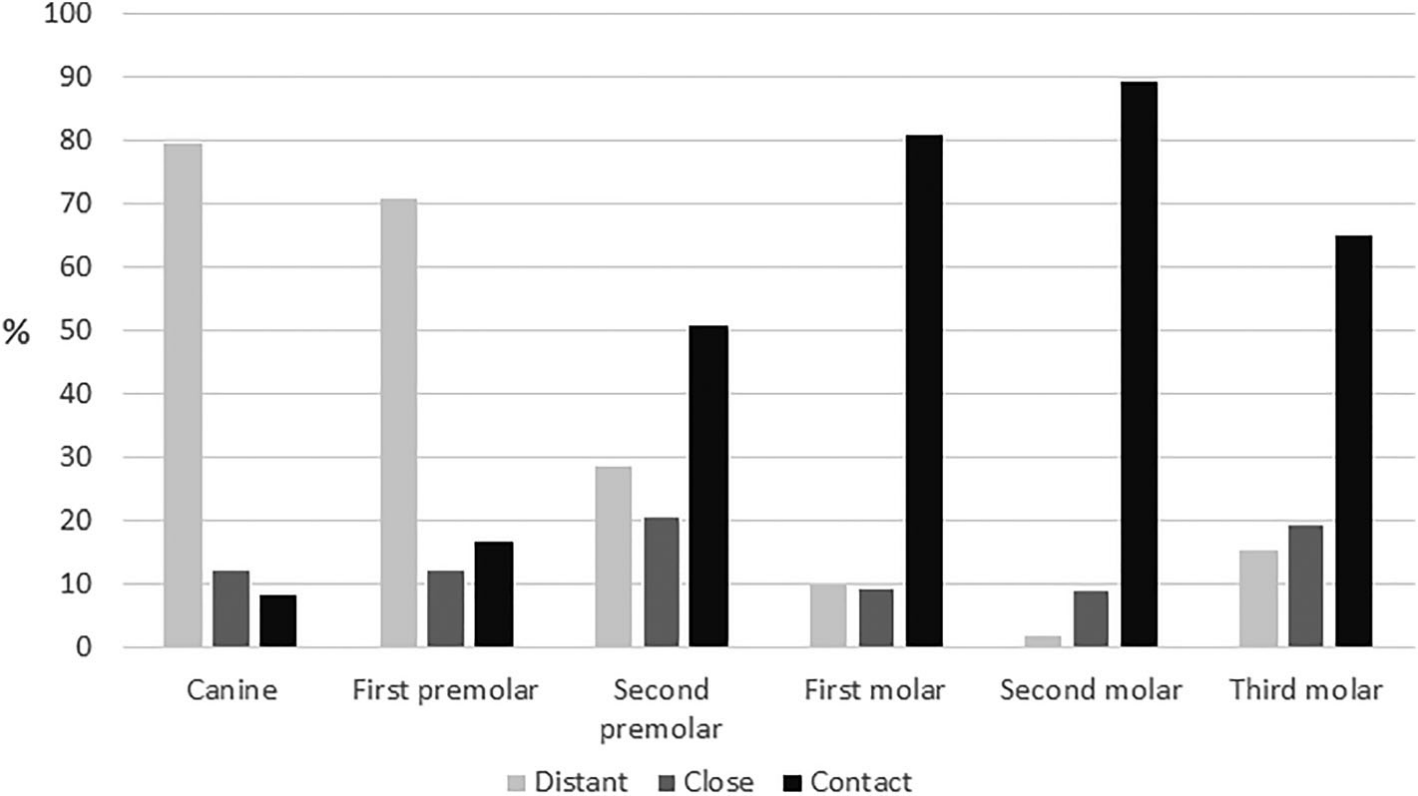
Overview of the anatomical relationship (distant, close or contact) in between posterior maxillary teeth and the maxillary sinus

Teeth most often in contact with the MS were the 2nd molar (89%) and the 1st molar (81%), although there was no significant difference between them (*p*‐value 0.19). The 3rd molar was in contact with the MS in 65% of the cases followed by 2nd premolars (51%), 1st premolars (17%) and canines (8%; Table 1, Figure 3). If a patient has one tooth in contact with MS, the probability is 82% that additional teeth are in contact with MS. The odds for being in contact with MS for 1st molars and 2nd molars are 344 and 909 times higher than for canines. The odds for being in contact with MS for 2nd premolars are 13 times higher than for 1st premolars. (Table 2).

**TABLE 1:** Anatomical relationship of canines and posterior maxillary teeth and the maxillary sinus on tooth and root level

Tooth category	Root category	Distant		Close		Contact	Total
		*n*	%	*n*	%	*n*	% *N*
Canine	Mono	202	79.6	31	12.2	21	8.3254
1st premolar	Tooth level	144	71	25	12.3	34	16.7203
	Mono	68	73.9	14	15.2	10	10.9 92
	Buccal	89	80.2	13	11.7	9	8.1111
	Palatal	78	70.3	9	8.11	24	21.6111
2nd premolar	Tooth level	57	28.6	41	20.6	101	50.7199
	Mono	53	28.9	38	20.8	92	50.3183
	Buccal	6	37.5	2	12.5	8	5 21
	Palatal	4	25	4	25	8	5 21
1st molar	Tooth level	20	9.8	19	9.3	165	80.9204
	Mesiobuccal	33	16.2	32	15.7	139	68.5204
	Distobuccal	34	16.7	38	18.6	132	64.7204
	Palatal	29	14.2	25	12.2	150	73.5204
2nd molar	Tooth level	3	1.7	15	8.9	151	89.3169
	Mesiobuccal	4	2.37	22	13	143	84.6169
	Distobuccal	14	8.28	26	15.4	129	76.3169
	Palatal	22	13.2	30	17.7	117	69.2169
3rd molar	Tooth level	7	15.2	9	19.5	30	65.2 46
	Mono	5	17.8	6	21.4	17	60.7 28
	Mesiobuccal	2	11.1	5	27.7	11	61.1 18
	Distobuccal	2	11.1	6	33.3	10	55.5 18
	Palatal	5	27.7	4	22.2	9	50 18

**TABLE 2:** Table over significant differences between tooth categories and their relationship to maxillary sinus

Relationship to sinus	Tooth category	Odds ratio	*p* Value
Distant	canine‐2nd premolar	19.4198	0.0001
Distant	canine‐1st molar	94.0307	0.0001
Distant	canine‐2nd molar	668.0656	0.0001
Distant	canine‐3rd molar	56.4372	0.0001
Distant	1st premolar‐2nd premolar	10.7749	0.0001
Distant	1st premolar‐1st molar	52.1722	0.0001
Distant	1st premolar‐2nd molar	370.6711	0.0001
Distant	1st premolar‐3rd molar	31.3137	0.0001
Distant	2nd premolar‐1st molar	4.842	0.0005
Distant	2nd premolar‐2nd molar	34.4012	0.0001
Close	2nd premolar‐1st molar	2.518	0.0194
Close	2nd premolar‐2nd molar	2.6373	0.0268
Contact	canine‐1st premolar	0.3102	0.0323
Contact	canine‐2nd premolar	0.0238	0.0001
Contact	canine‐1st molar	0.0029	0.0001
Contact	canine‐2nd molar	0.0011	0.0001
Contact	canine‐3rd molar	0.0064	0.0001
Contact	1st premolar‐2nd premolar	0.0766	0.0001
Contact	1st premolar‐1st molar	0.0094	0.0001
Contact	1st premolar‐2nd molar	0.0037	0.0001
Contact	1st premolar‐3rd molar	0.0206	0.0001
Contact	2nd premolar‐1st molar	0.1231	0.0001
Contact	2nd premolar‐2nd molar	0.0483	0.0001
Contact	2nd molar‐3rd molar	5.556	0.0163

The MB root of the 2nd molar is in contact with the MS in 85% of all cases, followed by the DB root of the same tooth (76%), the palatal root of the 1st molar (74%) and the P root of the 2nd molar (69%). For the 2nd molar, there is a significant difference when comparing the individual roots in contact with MS. The MB root is 1.1 times closer to the MS than the DB root (*p* = 0.0004) while the DB root is 1.2 times closer to the MS than the P root (*p* = 0.006). When comparing MB and P roots, the MB root is 1.2 times closer to MS (*p* = 0.0001). For the 1st molar, the percentages of roots in contact with the MS for the individual roots are 69% for the MB root, 65% for the DB root and 74% for the P root. There is a significant difference between P root, and MB or DB roots of the 1st molar. Yet, buccal roots do not significantly differ (*p* = 0.2). The P root is 1.14 times closer to the MS than the DB root (*p* = 0.0001) and 1.05 times closer than the MB root (*p* = 0.02).

A fifth of all canines were found to be situated less than 2 mm from the MS, with 8% being in contact with the MS.

### 3.2 Age and gender

There was no significant gender effect on the relationship between PMT and MS. The percentage of PMT in contact with MS were 45% for women and 49% for men. The percentage of roots in contact with MS were 53% for women and 51% for men. The proportion of patients with at least one tooth in contact with MS are 91% for women and 86% for men.

The relationship with MS for different tooth types and their different roots was also compared between age groups. The present study found no significant age‐effect on the relationship of PMT and MS in the contact relationship. There was no significant difference in the relationship of PMT and MS between different age groups (percentage of teeth in contact with MS ≤20 years 60.9%, 21–40 years 48.8%, 41–60 years 46%, >60 years 39.7%). Also, no significant differences on root level were found between the age groups, except for the DB root of the 2nd molar in a distant relation to the MS. The only noticeable fact was that a distant relationship was 4.9 times more common for the 41–60 years old, in comparison to the >60 years old age group (*p* = 0.036).

## 4 DISCUSSION

Knowledge of the anatomical relationship between MS and PMT is a necessity when assessing inflammatory response related MS problems with possible dental cause (Mehra & Murad, 2004; Vestin Fredriksson et al., 2017). The present study compared the relationship between the MS and roots of canines and PMT on CBCT and found that 1st and 2nd molars are the teeth most often in contact with MS. Radiological examination of the MS and surrounding structures can be accomplished with a variety of modalities. Earlier studies concluded that two‐dimensional imaging such as panoramic and intra‐oral x‐rays are unreliable methods for the evaluation of such relationship in detail (Kirkham‐Ali et al., 2019; Lofthag‐Hansen et al., 2007; Lopes et al., 2016; Shahbazian et al., 2014; Shahbazian et al., 2015; Sharan & Madjar, 2006). CBCT has been used in anatomical analysis and measurements and is considered as an accurate and reliable method (Ganguly et al., 2016; Howe, 2009).

This study focused on the closest distance from the radiological apex to the MS. The closest distance is of interest in risk assessment and treatment planning in upper posterior teeth. To make the measurements more comprehensible, the values in this study were subdivided into three groups. Previous studies have a different structure when grouping the measures. The grouping of the measures in the previous study were selected for a more comprehensive approach in understanding the relationship between canines, PMT and MS when diagnosing sinusitis with a dental cause. For 1st premolars, earlier research has concluded that the majority of the apices are outside the MS, 97.7–99.3% of buccal roots and 92.8–92.9% of the palatal roots (von Arx et al., 2014). These values are in line with the result of the present study where 96% of the 1st molars are outside the MS (Table 1).

The close relationship between the apex of the canine and the MS suggests that the maxillary canines needs to be considered when assessing a sinusitis with a possible dental cause. The relationship between the canine and the MS has not been investigated as thorough as the relationship of upper posterior molars and premolars. In the present study, 8.3% of the canines are in contact with the MS. Moreover, 20% of the maxillary canines are located less than 2 mm from the MS and may cause sinusitis of dental origin.

First molars in contact with MS (Table 2) were in the present study considerably more prevalent than in an earlier study that measured the number of 1st molars protruding into the MS (Nino‐Barrera et al., 2018). Although, it is important to notice that the way of grouping the measurements differ from current studies. The present study includes protruding roots and roots that touch the MS in one group, while other studies separate them. The result of previous studies assessing molars roots in contact with MS; 20–63% for second upper molars, 25–84% for first upper molars (Kilic et al., 2010; Nino‐Barrera et al., 2018; Shahbazian et al., 2014). Our results are in agreement with previous studies that have used CBCT for assessing the anatomical relationship between MS and roots of PMT (Estrela et al., 2016; Jung & Cho, 2012; Kilic et al., 2010; Pagin et al., 2013; Yoshimine et al., 2012). Although, a Brazilian study has found that the palatal root of the first molar is the root most often protruding into the MS (Nino‐Barrera et al., 2018). Possible reasons for the difference in results between studies may be related to sample selection (ethnicity and selection of cohort) as well as methodological differences (approach to subdivide and perform measurements). Previous studies in this field have assessed the relationship of the roots and MS in different ways: roots outside of the MS, roots touching the MS and/or roots protruding into the MS (Nino‐Barrera et al., 2018; Shahbazian et al., 2015; von Arx et al., 2014). Because of the close relationship between molars and the MS, it is 11 times more likely that molars are involved in sinusitis of dental origin compared to premolars (Maillet et al., 2011).

Approximately a fourth of the 2nd and 3rd molars have an uncommon root configuration. The relationship to MS for teeth with uncommon root configuration was not assessed in this study. Only roots with a normal anatomy were included in this study, for a more comprehensible interpretation of the results.

Significant age‐ and gender‐differences could not be reported in the present sample. The only noticeable fact, was an almost five times more common distant relationship for the 41–60 years old in comparison to the >60 years old age group (*p* = 0.036). The influence how aging and dental status influence the sinus volume are not fully understood (Bornstein et al., 2019; Velasco‐Torres et al., 2017). Although, a previous study found that the MS volume is decreasing with higher age irrespective of dental status (Bornstein et al., 2019). Furthermore, absence of adjacent teeth leads to a shorter distance between PMT and MS (Gu et al., 2018). Although, the distance between maxillary molar roots and MS are reported to increase with age (Gu et al., 2018; Pei et al., 2020). As for the gender‐effect, another study reported that males have a closer relationship between first premolars and MS than females (Ok et al., 2014). In future studies, both age‐ and gender‐effect should be further addressed in studies with bigger sample sizes.

The most important message to clinicians is that most of the first and second molars are in contact with the MS. This is important knowledge in endodontics, especially when performing apical surgery or in complicated extractions. Knowledge of the relationship between PMT and MS is also valuable when investigating the cause of odontogenic sinusitis. The closer relationship between the apex and the MS, the greater is the risk of a spreading infection from a tooth into the MS (Mehra & Murad, 2004).

## 5 CONCLUSION

The present data suggest that 1st and 2nd upper molars are the teeth most often in a close relationship to MS. The root in closest relationship to MS is the mesiobuccal root of the 2nd molar followed by the distobuccal root of the 2nd molar and the palatal root of the 1st molar. Upper canines need to be taken into consideration for their potential sinus relation, surely when investigating sinusitis with a possible dental cause.

## ETHICS STATEMENT

The study conforms to the ethical standards of the Declaration of Helsinki.

## CONFLICT OF INTEREST

The authors declare there is no conflict of interest.

## AUTHOR CONTRIBUTIONS

**Tobias Regnstrand:** Study design, data analysis, data interpretation, manuscript drafting, approval of final version. **Andres Torres:** Study design, data acquisition, data analysis, manuscript revising, approval of final version. **Eline Petitjean:** Study design. Data acquisition, manuscript revising, approval of final version. **Paul Lambrechts:** Study design, manuscript revising, approval of final version. **Daniel Benchimol:** Study design, data interpretation, manuscript revising, approval of final version. **Reinhilde Jacobs:** Study design, data interpretation, manuscript revising, approval of final version. All authors are agreed to be accountable for all aspects of the work.

## Data Availability

Data available on request from the authors
